# A qualitative investigation of infusing products with service via strategic alliances among SMEs: a case of servitization

**DOI:** 10.1007/s11628-023-00530-2

**Published:** 2023-03-16

**Authors:** Diogo Pombo, Mário Franco

**Affiliations:** Department of Management and Economics, CEFAGE-UBI Research Center, University of Beira Interior, Estrada do Sineiro, 6200-209 Covilhã, Portugal

**Keywords:** Servitization, Strategic alliances, SME, Motives

## Abstract

This study aims to understand the role of strategic alliances in successful SME servitization. A qualitative approach was adopted, elaborating a script for semi-structured interviews to be applied to some SMEs. Based on content analysis and using NVivo software, it is concluded that servitization and alliances are two strategies that are connected, with a complementary relation, through the share of resources and knowledge, creating competitive advantages and adding value to business models, while also improving the final client’s experience. This study intends to contribute more insights to provide SME owners–managers with the results of combining the two strategies analysed here.

## Introduction

In recent years, the business world has witnessed major technological changes and a consequent increase in competitiveness (Ruiz-Martín and Díaz-Garrido 2021). Firms need to adopt strategies to face the volatility of the market, creating an attacking position with regard to new opportunities and a defensive one with regard to potential threats (Machado-da-Silva and Fernandes 1998). In this context, there has been a sharp increase in firms’ adoption of strategies to introduce services in their business models, due to their potential to create innovation and competitive advantages (Konsti-Laakso et al. 2012), as well as forming new trusting and loyal relations with customers (Johansson et al. 2019), and recovering competitiveness and stimulating innovation (Gebauer et al. 2021). When introducing services, the focus is on creating value, since in the most developed economies, it is more frequent for firms to compete based on the value delivered than based on costs (Kinnunen 2011). Although the literature presents no common standard structuring this situation, various researchers call the process of introducing services as “servitization” (e.g., Vandermerwe and Rada 1988; Kowalkowski et al. 2017).

The concept of servitization was first presented by Vandermerwe and Rada (1988), who found an increasing tendency to introduce services in firms’ business models, as a means to increase productivity and market power, and differentiate themselves from other organisations of the same size. Later, various scholars deepened this concept as a way for firms to give their products a new identity (Morelli 2003) and deepen new business models (Kowalkowski et al. 2017). Servitization aims to determine a new value proposition for the business model, as it refers to the transformation of product-centric business models into service-oriented business models, which can bring companies, strategic and competitive benefits as well as improve innovation processes (FFMS 2019). This strategy allows companies to increase their revenues by offering integrated or combined solutions between products and services in order to give more value to their clients (Xu et al. 2021).

Servitization can be explored as a tool to accelerate the innovation allows firms to improve their competitive advantages, by presenting a higher degree of differentiation and reducing the probability of being imitated by competitors in the same branch/sector (Gebauer 2008). The firms are creating something new and different, offering more efficiency to the product and giving more value to their customers, increasing the loyalty rate (Coreynen et al. 2017). By changing their strategy, companies can discover new sources of income when they expose themselves to the market, i.e., they can receive new insights, for example, from their own customers, which will allow them to understand their needs and explore it more maintaining their existing offerings (Khanra et al. 2021).

Therefore, introducing services can be especially relevant in small and medium-sized enterprises (SME) (Man et al. 2002). This firms segment has a very significant role in economic and social development, particularly in the European Union (EU), since it is considered that “*98.9% of firms in the EU’s non-financial market economy are small in size, employing around a hundred million workers”* (European Parliament 2020). SMEs are predominant in most economies and they represent an important role, not because they are numerous, but because of what they can represent, like, important source of jobs, first step in many entrepreneurs’ career, launchers of new ideas and new work processes and they have capacity of counter-balance the monopoles and oligopolies reducing their capacity of controlling the market (Savlovschi, and Robu 2011).

SMEs, generally are characterised by their shortage or even lack of resources (Franco and Haase 2015; Cao and Zhang 2011), this uncertainty and unpredictability are even greater. As the external surroundings are determining factors for SMEs, strategic alliances become particularly relevant for these firms (Beckman et al. 2004). Moreover, their resources constraints make SMEs more eager to establish strategic alliances with other firms as a via of Infusing Products with service. Therefore, implementing servitization can bring advantages and overcome difficulties in sustaining innovation, due to the lack of resources (Rapaccini et al. 2019). In isolation, SMEs cannot achieve the sustainable profitability of a service firm, since they do not have the necessary financial and management resources (Gebauer et al. 2012).

In these circumstances, the literature shows that SMEs can choose to form strategic alliances as a way to obtain the resources they lack and to overcome the above barriers to servitization (Klotzle 2002; Barbosa et al. 2009; Iturrioz et al. 2015; Rapaccini et al. 2019). Through strategic alliances, SMEs can increase the management capacity of servitization, in order to be successful and efficient (Carneiro 2021). However, the existing literature on servitization and the reasons leading SMEs to form strategic alliances to implement this strategy is still limited (e.g., Iturrioz et al. 2015). Indeed, Rapaccini et al. (2019) show this gap in the literature and make a case study of Italian firms showing that the firms analysed achieve successful servitization through strategic alliances. The literature contains few studies exploring the formation of strategic alliances as a response to the complexity of servitization for SMEs (e.g., Klotzle 2002; Barbosa et al. 2009; Konsti-Laakso et al. 2012; Kowalkowski et al. 2013; Rapaccini et al. 2019).

This reveals the need for study in greater depth to understand in what way servitization becomes more viable when SMEs decide to form alliances with other firms, highlighting the reasons for this type of relation. This study aims to show how strategic alliances formed by SMEs can be a viable strategy for more successful servitization, allowing the development of better products and services and increased market participation. It seeks to answer the following research question: *How infusing products with service *via* strategic alliances among SMEs, aiming for successful servitization?* Based on the two strategies explored here—servitization and strategic alliances, the study intends to contribute to identifying and understanding how SMEs deciding to form alliances to implement a successful servitization process.

The article is structured as follows. The Introduction is followed by the Literature Review about servitization and strategic alliances. The next section presents the method and then the Discussion of the Results, ending with final considerations and the implications of the study.

## Literature review

### Servitization and reasons for forming strategic alliances

In developed countries, services have become an important pillar of their economies and have allowed firms to compete in producing value rather than producing cost, also considering market demand and clients’ increasingly complex needs (Bikfalvi et al. 2013). By understanding clients’ needs, firms are able to improve and increase their economic results (Gebauer et al. 2012).

The integration of services in business models, according to Oliva and Kallenberg (2003), has three fundamental aims: to generate greater profit compared to a business model focused only on the product, responding to clients’ more complex needs and as sources of competitive advantages, as these are more difficult to imitate. However, mainly in the SME context, to integrate services, they must have the capacity to organize people, resources, technology and information (Chalal et al. 2015). Khanra et al. (2021) find that initially SMEs seek to combine simple services with their existing products, but they are not able to generate sufficient gains to sustain that strategy. This is where servitization arises.

Among the different organisational strategies, in recent years the introduction of one or more services to business models has been a growing trend in diverse sectors, due to the potential to create innovation and competitive advantages (Konsti-Laakso et al. 2012). However, according to Kowalkowski et al. (2017), there is no common pattern of procedures structuring this phenomenon, which many researchers class as “servitization” (Vandermerwe and Rada 1988; Baines et al. 2009; Rapaccini et al. 2019), also being interpreted as “service infusion” (Kowalkowski et al. 2017) or even New Product Development (NPD) (Marzi et al. 2020). Others follow the vision based on a Product-Service System (PSS), considering servitization as a special case (Chalal et al. 2015) or “*Smartization*”^1^ (Kaňovská and Tomášková 2018). The literature also contains authors who, despite not referring to the topic of servitization, cite the term of “service infusion”, as is the case of Oliva and Kallenberg (2003).

Morelli (2003) defined this strategy as an evolution of product identity. Baines et al. (2009) argued that servitization can be considered a strategy to innovate an organisation’s capacities and processes which helps to improve or create mutual value through a change from product sales to sales of product service systems. Kinnunen (2011) states it is a question of increasing added value by adding services to the supply, foreseeing a strategic need for change in the organisation.

Integrating services gives new business opportunities, as firms can carry out activities for clients or introduce services combined with products (Almeida et al. 2011). It is a combination of tangible products and intangible services in a product or service package (Bikfalvi et al. 2013). According to Rapaccini et al. (2019), it is a strategic transformation in a firm that, deliberately or in an emergency, introduces service elements in its business model, no longer being centred on the product, while FFMS (2019) say it is a transformation of the business model oriented to service. Martin et al. (2019) conclude that servitization is a means to achieve and continuously improve results of value, by introducing services.

Servitization induces a continuous change in the corporate culture of the whole firm, which needs a service-oriented vision, through internal strategies for the commitment of all (management and collaborators), and with clients’ participation in the conception, production and consumption of products and services (Dubruc et al. 2014).

Therefore, in today’s globalized world, the formation of alliances can have a positive effect on innovation capacity and allow an advantage over competitors, through improvements in organisational and financial performance, besides increasing the capacity to provide suppliers and purchasers with quality (Yang et al. 2015). For SMEs, servitization can be more easily achieved by forming strategic alliances.

Strategic alliances are voluntary agreements between firms, involving exchanges, sharing or co-development of products, technology or services (Gulati 1998). Das and Teng (2000) state these are cooperative agreements aiming for competitive advantages arising from technological progress and increased market competitiveness, following a perspective based on resources and strategic needs. Alliances involve at least two firms that remain independent, but which contribute continuously and share tasks and benefits (Todeva and Knobe 2005). They are a potential source of competitive advantages, cost reduction and keeping the focus on essential competences (Barbosa et al. 2009).

Strategic alliances allow firms to focus on their competences while relying on their partners in other areas where they are less well-equipped (Swoboda et al. 2011). These cooperative relations establish commercial relations, and share competences and resources in order to improve efficiency and create value in business models, i.e., value creation is the result of actors’ interactions (Kowalkowski et al. 2013). Citing the study by Contractor and Lorange (1998), from the analysis of Cárdenas and Lopes (2006), these cooperative agreements can give firms seven benefits, among them risk reduction, cost reduction, exchange of knowledge, less competition, operations within borders and possible entry to other markets.

According to Todeva and Knobe (2005), the continuous contributions of these cooperative relations bring benefits for partners in the form of tangible and intangible assets in one or more strategic areas, such as product and technological development, co-specialization of products and services or overcoming barriers. The benefits of an alliance indicate greater flexibility and access to resources without the risk of inherent responsibilities, besides allowing increased competitive advantages (Barbosa et al. 2009).

The study by Lin and Lin (2012) presents the factors that motivate SMEs to form alliances, these being growing competition, technological complexity, and dependence on external resources, but the existence of a network of understanding is not enough for a successful strategy. It is necessary to define objectives and mutual tasks.

According to Howard et al. (2016), learning from a partner gives common and individual benefits. For example, when the knowledge acquired is applied in the firm’s own operations, when it develops collaborative management skills or when other partners’ valuable methods and models are made use of. For O’Dwyer and Gilmore (2018), the concept of capturing value is closely linked to the ethos of strategic alliances, as they can focus on the creation of value (developing new products using different approaches) or on capturing value (intangible value, learning process, overcoming challenges).

Therefore, alliances stimulate value creation, allow access to information, knowledge, production and distribution capacities and access to other technology, lower transaction costs, help to respond to market pressure, increase competitive advantages, legitimacy and reputation (Niesten and Jolink 2020). Innovative activities generate risks, since the economic value is difficult to predict. Here, Spieth et al. (2021) conclude that involving strategic partners in value creation and innovation helps firms to control risks and costs while receiving additional resources, knowledge and technology. Also for Spieth et al. (2021), through alliances, firms overcome inflexibility or even inertia, as they become more able to improve knowledge and have more resources at their disposal.

### Strategic alliances as a tool for servitization

Various authors propose that models based on cooperative relations (alliances) can offer more benefits with greater effectiveness to models combining products and services (Khanra et al. 2021). Strategic alliances help SMEs in their servitization processes, as this is a way to share the costs, resources, knowledge and capacities required by this strategy (Rapaccini et al. 2019).

A firm that decides to implement a servitization strategy needs to adopt a service-oriented culture (Kowalkowski et al. 2013; Dubruc et al. 2014) and a client-oriented culture (Perin 2001). However, due to their limited resources (Gebauer et al. 2012; Wang et al. 2016), firms are not able to develop this new culture of innovation (Oliva and Kallenberg 2003; Gebauer 2008).

An SME needs to develop new organisational, operational and commercial capacities (Chalal et al. 2015), i.e., create innovation processes (O’Dwyer and Gilmore 2018; Xu et al. 2021), as is the case of introducing services. Part of the literature agrees that firms reduce risks and achieve better results as they provide services (Benedettini et al. 2015). Innovation is the key question for any type of firm, but especially SMEs (Valentim et al. 2013) and the capacity to innovate gives advantages in rising above the competition (Yang et al. 2015).

Innovation and business networks are essentially linked, and an innovation is the fruit of interactions between various actors (Ojasalo 2008). Therefore, alliances are conceived as useful approaches to share and reduce costs (Das and Teng 2000; Cárdenas and Lopes 2006), obtain strategic resources (Klotzle 2002; Hofer 2020) and knowledge to complement activities (Grant 1996; Spieth et al. 2021), which are very relevant factors for innovation (Konti-Laakso et al*.* 2012). The strategic alternative for SMEs is to combine resources and skills with other entities, supplying new products and services (Valentim et al. 2013). Rapaccini et al. (2019) argue that SMEs can form alliances with other SMEs or larger firms, as a way to overcome the aforementioned barriers to innovation, exploit better the scarce resources available and also facilitate the growth of their service business, as alliances can increase the chances of successful, sustainable servitization processes.

Cooperation and coordination with external partners can make it easier for firms to respond to external factors and allow value creation (Gulati 2007). Alliance formation does not only favour access to new, external resources, but also new competitive advantages through sharing experiences and knowledge (Rodrigues et al. 2021).

Strategic alliances are an alternative to the internationalization of a product or service, through the firm’s own production, the acquisition of markets or jointly with partner firms (Das and Teng 2000). In the context of strategic alliances, value creation and competitive advantages are the result of actors’ interactions (Kowalkowski et al. 2013), with it being crucial to cultivate good relations at the beginning of a service infusion process, when firms’ infrastructure is less prepared (Gebauer 2008). This occurs in the case of servitization, when firms decide to expand their business models in order to deliver more value to the final consumer (Ruiz-Martín and Díaz-Garrido 2021).

Introducing new services and exploiting dynamic resources to gain new market opportunities can occur through strategic alliances (Khanra et al. 2021), and according to Rapaccini et al. (2019), the literature is consensual that the stimuli of these relations help to face up to the challenges of implementing services. The literature also says that servitization is complex, as it needs skills in identifying, assessing and managing long-term risks in supply flows (Benedettini et al. 2015). Therefore, strategic alliances emerge as an important strategic choice in the current climate (Franco 2011).

According to Chalal et al. (2015), it is not enough to define a new business model based on services. There must be deep internal transformation, through a re-organisation of internal strategy. The most direct objective of servitization is not only to obtain higher profits, but also to strengthen the product-related business (Lexutt 2020). Servitization is a new way for manufacturers to create value, through new offers proposed to clients (Xu et al. 2021), with a view to raising the client’s value and experience (Ruiz-Martín and Díaz-Garrido 2021) and it can also ensure firms’ future competitiveness (Khanra et al. 2021). According to Kharlamov and Parry (2021), servitization captures additional value by providing complete packages with a combination of goods and services, concentrated on just one service.

In sum, Table 1 show the extant literature identified following the leans an structure of Zahra and George (2002).

**Table 1:** Conceptualizattion and operationalization issues

Study	Theoretical lens	Methodology	Sample/data	Independent variables (IVs) and dependent variables (DVs)	Results	Contributions
Oliva and Kallenberg (2003)	Transformation and Organizational learning	Qualitative	11 German capital equipment manufacturers	Manufacturing production, Transformation effort, Services operations isolated and adopt horizontal service delivery structure (IVs), service introduction result (DVs)	Theoretical model of the transformation patterns followed by firms that had attempted the transition	Identification of the implications for a transition to service organization in the manufacturing firm context
Baines et al. (2009)	Concept of servitization	Qualitative	58 papers reviewed in detail	–	Summary of the currently available literature on the subject of servitization	Review of the concept of servitization
Kinnunen (2011)	Organizational and culture learning	Qualitative	1 company with 20 different employees from different organizational levels	–	Study of servitization in manufacturing companies and framework	Theoretical and practical framework that concentrates on organizational culture and configuration for servitization
Rapaccini et al. (2019)	Strategic management	Qualitative	1 consortium with 19 SMES	Strategic alliance (IV), Servitization (DV)	The consortium has being a driver of servitization strategy and business have grown rapidly	Strategic alliances among SMEs can contribute to increase the chances of a successful and sustainable servitization processes
FFMS (2019)	Business Model Innovation (BMI)	Qualitative	Cases from literature	Innovation (IV), product firms (DV)	Services can bring value to the customers and be a channel of information to improvements	Conceptual framework that connects Servitization and Industry 4.0
Khanra et al. (2021)	Bibliometric analysis	Quantitative	275 articles from journals rated 3 or more in ABS	–	Comprehensive view of the extant literature of servitization addressing important thematic areas	Review and bibliometric analysis of servitization research
Xu et al. (2021)	BMI in emerging economies	Qualitaitve	2 leading piano manufactures	Innovation, structure and governance (IVs), manufactures companies (DVs)	Innovation, structure, and governance can overcome servitization barriers	Framework of BMI, mechanisms of dynamic capabilities to promote BMI, and importance of implementation business model with ambidexterity
Ruiz-Martín and Díaz-Garrido (2021)	Resource-based view, Game theory, and Transaction cost economics	Quantitative	93 papers	–	Combination of different approaches of servitization	State-of-the-art on the theory of servitization

## Methods

### Type of study and case selection

The main aim of this research is to understand the role played by strategic alliances in SMEs’ servitization, and how they affect service implementation positively and/or negatively, identifying the motives for these two types of strategy. To do so, a qualitative approach was adopted, through multiple case studies.

The qualitative focus of a study aims to present a descriptive character, i.e., identify the main characteristics and situations to allow researchers to measure opinions and seek to understand the interactions presented in the samples of the universe studied (Freitas and Jabbour 2011). Qualitative research also allows the use of a variety of documents and procedures for data analysis, obtaining information and revealing new aspects of a topic or problem, resulting in the extraction of relevant content for research (Kripka et al. 2015).

The case study allows understanding, in a real context, of the nature of a phenomenon that is not under control, in most cases being contemporary, social contexts (Yin 2015). As a particular strategy of qualitative methodology, Toledo and de Farias Shiaishi (2009) indicate that the case study method helps the researcher to understand how certain phenomena are established in some firms. The same authors found that better results are obtained in case studies, as these imply greater analysis of the details of the relations between individuals and firms, and of changes in the environments the actors belong to.

More precisely, for this study, multiple cases were chosen (Kinnunen 2011; Hofer 2020), of three Portuguese SMEs and three Spanish SMEs. The criterion for selecting these SMEs was being classified as SMEs by the European Commission. Subsequently, these firms should carry out their activity in these two countries, i.e., these firms were chosen for convenience and because of having easy access to information. Another criterion for selecting these SMEs was that they should belong to different sectors of activity, so as to give greater variety in the results. As the last criterion, the selected firms should be in a strategic alliance with at least one other service SME. In addition, after the pandemic Covid-19, the European Commission advised the SMEs to adopt necessary measures to fight against the incoming crises, even though they created a recovery packaging that should boost the economy of the European countries and launched funded projects, they also encouraged the creation of new corporations between traditional SMEs and SMEs with technological knowledge (European Innovation Council and SMEs Executive Agency 2021).

Despite not being the aim of this study, including firms from two countries allows a comparison, according to Širá et al. (2020), with different economic indices. According to *Información Estadística Europea* (INE Spain 2022), in 2021, Portugal’s gross domestic product (GDP) was 211,278 million euros, while the Spanish GDP for the same year was 1,205,063 million euros.

As these are micro-firms, all the alliances formed are with regional partners, since this is more convenient for them, to perform their services and keep in close contact with their partners.

Table 2 presents a brief characterisation of these SMEs:

**Table 2:** Firm characterization. *Source*: Own elaboration

	Main activity	Years of activity	Types of service supplied	No. of collaborators	Average number of alliances	Allied firms’ activity
A	Joinery	20 years	Customized services, transport services (delivery and collection), installation services	3	2	Joinery services, provide the same service as Firm A
B	Public transport	5 years	Public transport, collective transport of children, private transport	4	4	Public transport services (taxi)
C	Monitoring services	2 years	Maintenance services, Repair services	5	2	Production, packaging and delivery of perishable products; marketing services
D	Photography and design services	15 years	Customized services	4	3	Design and production services
E	Clothing manufacture	5 years	Customized services, transport services (delivery)	12	1	Transport services—product delivery
F	Technology sales and services	10 anos	Maintenance services, repair services, customized services monitoring services	8	2	Software maintenance services

The data obtained from the six SMEs (cases) revealed that some firms provide more than one type of service. Besides producing joinery products, Firm A provides customized services, transport (delivery and collection of old and new objects) and installation. Firm B, being focused on transporting people, works in public transport, collective transport of children and private transport (for insurance agencies). As Firm C monitors food products, using electronic equipment, this can get damaged during transport, so the firm also has the service of maintaining and repairing equipment. Through its photographic and design services, Firm D supplies personalized catalogues, i.e., customized at each customer’s request. Firm E has different ranges of clothing, with some being customized and then delivered to the final customer. Finally, Firm F supplies a combination of almost all the services already mentioned, as it sells and repairs electronic equipment, produces customized software and carries out monitoring.

### Data collection and treatment

The interview is one of the most common data-collecting techniques in qualitative studies, as stated by Opdenakker (2006). Unlike questionnaires sent by mail, which have a low rate of return, the interview provides more wide-ranging answers, since participants are ready to speak about certain subjects (Selltiz et al. 1987). Therefore, for this study data were collected through semi-structured interviews with the owners–managers of the SMEs chosen, aiming to analyse the impacts and results of strategic alliances in these firms’ servitization, in a real context. This type of interview combines open and closed questions, letting the interviewees speak in depth about the topic proposed (Selltiz et al*.* 1987). According to Trinczek (2009), firms’ owners–managers and administrators are the agents/actors with the broadest view of the business. Therefore, the SMEs were asked for the interviews to be held with these agents.

For the interviews, a script with questions was clearly transcribed, so that the interviewee would easily understand what was asked. The questions were not taken from specific studies, but elaborated from gathering ideas from various studies, such as Hofer (2020), Kinnunen (2011) and Rapaccini et al. (2019).

This interview protocol was subject to a pre-test with three business-people who have been operating for a considerable number of years and have great business experience. Only a few terms were changed to help the future interviewees’ understanding of the script.

The participants were contacted initially by telephone and/or e-mail, to arrange a possible meeting face-to-face or online (call or video-conference). After determining their availability, six sessions were arranged for the interviews. These lasted between 35 and 60 min on average, two being held face-to-face, two by telephone and two by video-conferencing. Due to the Covid-19 epidemic, four of the six interviews were held at a distance, the other two being held at the interviewees’ places of work. The interviews took place in December 2021 and January 2022. Table 3 presents a brief description of the interviews:

**Table 3:** Characterization of the interviews. *Source*: Own elaboration

Interviewee	Post	Country	Academic qualifications	Gender	Years of activity (years)	Interview method	Interview length (min)^a^
A	Partner–manager	Spain	Secondary education	F	20	Telephone	50
B	Owner	Portugal	Diploma in Chemistry	M	7	Video-call	45
C	Partner–manager	Portugal	Master in Electronic Engineering and Computing	M	2	Video-call	60
D	Manager	Portugal	Degree in Marketing	M	15	Telephone	30
E	Manager	Spain	Degree in Economics	F	9	Face-to-face	45
F	Manager	Spain	Master in Computer Engineering	M	10	Face-to-face	60

^a^Approximately

Regarding the two interviews held over the phone, the interview script was sent first by e-mail, so that the interviewee would know the questions that would be asked. The answers were transcribed to a Word document and then dictated to confirm what had been said. In the two interviews held by video-conferencing, the script was also sent by e-mail and the answers were transcribed to the document and then confirmed by the interviewees. It is noted that one of the interviewees (Firm B) was willing, during the video-call, to show one of his contracts with an insurance agency for which he provides transport services. The idea was to explain how he carried out the service and the obligations of both parties in the alliance formed. This secondary document served to confirm the data collected about the firm (documentary analysis). Finally, the two face-to-face interviews were held on the premises of firms E and F, where a copy of the interview script was handed over. The answers were transcribed to the Word document and the interviewees could check and confirm their answers there and then. As a complement, Firm E presented a document that linked its alliance with the transport firm, which showed the terms established to perform the service, together with some bills for the service provided.

After holding the interviews and joining the main ideas expressed, the document with their answers was sent by e-mail to the respective interviewees, for them to confirm and/or correct their statements. Two of them provided more information and the other four confirmed that everything was as they had said. Finally, these documents were submitted to content analysis, using NVivo software, a platform to analyse qualitative data that helps to organize, analyse and reveal insights in unstructured data, such as interviews and open research questions.

Mozzato et al. (2016) showed the importance of this software and indicated the main advantages of using NVivo. They claim this software helps the researcher during the whole planning stage, in defining procedures, analysing data, developing theories and presenting credible and reliable results. The authors also mention the advantage of all the analyses made by the researcher being managed in a single place, with the possibility of importing any type of document and exporting it to other applications. In addition, this instrument makes trans-disciplinary studies possible. Botelho et al. (2017) mention even more advantages, stating that NVivo can establish relations between data quickly and efficiently, saving time and keeping close to the original data, as it helps to capture important characteristics of the documents.

Given these advantages, it was decided to use NVivo, proceeding to individual analysis of each interview, creating codes for the most cited variables and regrouping them subsequently in similar groups. The number of citations of each code corresponds, therefore, to the importance of the variables mentioned.

## Results and discussion

As already stated, this study aims to understand in depth the reasons for forming strategic alliances and how these contribute to successful servitization.

### Reasons for forming alliances

The first dimension/category studied here is the reasons leading SMEs to form a strategic alliance, in order to achieve better results/successful servitization. Indeed, strategic alliances are seen as a strategy supporting innovation, since they concentrate firms’ efforts in dynamic environments (Konsti-Laakso et al. 2012).

The results obtained indicate that the most cited reasons are *“Value Creation”, “Firm Development”* and *“Gain more time”.* Half of the interviewees said there was a great need to deliver a quality service to customers and that it was important to ensure that quality. Interviewee B, for example, who deals with passenger transport, states that “*the emergence of new market niches leads to looking for partners to do more work*”. Other three firms (B, D and F) also indicated that introducing new services to their business model led to them having more time, i.e., more time to concentrate and supply a good service, and also more time to improve the response time.

One of the reasons mentioned stood out as being different. Firm A, whose business model is focused on the production of craft products, stated that it only enters alliances occasionally *“When someone asks”,* such as a friend or acquaintance, and that alliance has more to do with helping than being helped, as according to the interviewee, “*almost all its activity is for one major client, Leroy Merlin in Málaga*”.

Two other reasons were mentioned *“Obtaining information”* and”*Obtaining material resources”*, by Firms C and B, respectively. Firm C, due to still being at an early stage of its activity, states that “*all the help and information that’s given is welcome*”. Firm B, due to working with vehicles, needs resources to be always available, which does not happen when a vehicle is in use or being repaired.

The reasons of *“Obtaining financial resources”* and *“Obtaining human resources”,* are mentioned by Firms C, B and F. Firm C has not been in existence long enough to have the capacity to invest as and when it needs, and Firms B and F need people with the right training (an appropriate driving licence and technological knowledge) because “*there was a growth in customer demand”.*

From NVivo, Table 4 shows the answers serving as an example for the dimension related to motives:

**Table 4:** Reasons for strategic alliances. *Source*: Elaborated by the author from NVivo

Dimension	Variable	Answers	No. of times cited	Firm
Motives	Value creation	“Need to deliver a complete, quality service” “Ensure quality”	3	D, E and F
	Firm development	“Improve the service” “Not to lose business niches and try to reach a wider market”	3	B, C and F
	Obtain information	“We have little experience”	1	C
	Obtain financial resources	“Lack of financial resources to be autonomous”	2	C
	Obtain human resources	“It’s a service that needs knowledge” “there was an increase in customers and work flow, which makes us look for help”	2	B and F
	Obtain material resources	“There was an increase in customers and work flow, which makes us look for help”	1	B
	Gain time	“Improve the response capacity” “Service needs time”	3	B, D and F
	Someone asks	“Because a friend or acquaintance working in the area asks for help”	1	A

As observed in the literature, different reasons explain why one firm decides to form an alliance with others, but three approaches stand out, the vision based on business resources (e.g., Hofer 2020), organisational learning theory (e.g., Grant 1996) and transaction cost theory (e.g., Das and Teng 2000).

To access, retain and develop auxiliary resources are some of the reasons leading SMEs to form an alliance (Hofer 2020). These reasons contribute to firms being able to increase their production capacity and have greater operational flexibility (Todeva and Knobe 2005). To obtain more material, human and financial resources are answers given by half the interviewees (Firms B, C and F), since these resources, when shared, are fundamental factors to achieve common and individual objectives, and subsequently determine alliance success and define a firm’s position in the market (Klotzle 2002).

The reduction of production and transaction costs aims to optimize the flow of goods and services, minimizing the costs associated with the business activity (Das and Teng 2000). Although none of the interviewees mentioned cost reduction directly as a reason for forming an alliance, the search for financial resources and improved services are variables that, indirectly, affect cost reduction. Indeed, governance mechanisms that help to establish task coordination (Das and Teng 2000) will contribute to developing business and saving on expenses.

Finally, the last theory mentioned most in the literature is organisational learning theory, which is explained by the transfer of knowledge, and applies greatly to Firms C and F, which seek information for business development and the technological knowledge shared by partners’ qualified collaborators. Implicit and explicit knowledge, mentioned by Grant (1996), are important for two firms to be able to develop their business models. Gaining more knowledge or learning increases the possibilities of improving competitive positioning in the market and consequently creating more value.

### Results of servitization arising from alliances formed by SMEs

Before going on to analyse the results that strategic alliances add to firms practising servitization, the interviewees were also asked about the results they drew from adding one or more services to their business models.

The answers obtained could be divided into three results: “*Value creation”, New sources of income”* and* “No results”.* In relation to the first result—*“Value creation”*, with servitization being a strategic change that includes overcoming various challenges (Kinnunen 2011), its aim is to generate new forms of value for the final customer (Xu et al. 2021). Answers given by Firms C, D, E and F confirmed there was an increase in the quality of service and supply, adding value to the final product.

As for the second result—*“New sources of income”*, according to Khanra et al. (2021), firms should have the capacity to capture new market opportunities in parallel with their operational functioning and maintaining existing supply. In this respect, Firms B, C and F managed, respectively, to *“provide more services and … increase invoicing”;* “*open up more branches”* and *“provide a diversified portfolio”*.

Finally, the only different result was obtained from Firm A—*“No results”*. According to the manager, from beginning her activity, besides supplying joinery products, they always provided *“delivery, installation and made-to-order work”*, so no different result is indicated as no differences were noted.

Innovation is one of the most important key elements for successful servitization, and it has significant positive impacts on changes to business models (FFMS 2019; Johansson et al. 2019). As mentioned previously in the literature, strategic alliances allow SMEs to overcome barriers to innovation (Rapaccini et al. 2019). Spieth et al. (2021) strongly recommend that firms should follow collaborative innovation strategies to organize the development of new business models requiring firms to be open to new practices and insights. However, Costa et al. (2021) state that the innovation process implies a logical sequence between knowledge and economic performance, with this conclusion being confirmed by the answers received in the interviews—*“It filled gaps”* (Firm C); *it facilitated the process that led the business to exploit new services”* (Firm D); and *“it automated production processes”* (Firm E). So it can be argued that strategic alliances have a positive effect on successful servitization, which is an innovation strategy.

Innovation processes are linked to value creation processes (Yang et al. 2015). Based on the study by Khanra et al. (2021), business models can gain new insights through the interactions of cooperation networks, combining benefits of the product and the service more effectively than traditional product-centred models. This statement agrees with revelations in the interviews, as strategic alliances allowed “*Value creation”* in these firms, both in adding value to the final product (e.g., Firm E) and in improving customer satisfaction (e.g., Firms B and C). Khanra et al. (2021) also indicate that when companies emphasize service differentiation, the capacity to understand clients’ needs increases. The study by Hofer (2020) also concludes that to face the globalized world, firms need to differentiate themselves. Once again, this information is similar to the results obtained in the interviews, particularly in Firms B, D and F, which improved their business models and increased invoicing through providing a more “*differentiated and diversified”* portfolio*.* Therefore, strategic alliances appear to contribute to value creation for firms, providing more differentiated and diversified products and services compared to the competition.in recent years, researchers have turned to focus on SMEs’ competitiveness and how this has increased substantially (e.g., Man et al. 2002). With servitization also being a strategy to improve a firm’s competitiveness in innovation processes (Khanra et al. 2021), strategic alliances emerge as a strategic means to achieve new product and service development, modelling their competitiveness on the market. The “*competition”* is indicated by the interviewees as a barrier to business development, either because they have more experience (Firm C) or because they compete with disloyal prices (Firm B). Here, strategic alliances contribute to lessening the effects of competitiveness on SMEs, through forms of cooperation to seek new business opportunities, access to new markets, sharing experiences, knowledge and resources, increasing the firm’s competitive advantages (Rodrigues et al. 2021).

Since SMEs do not have enough resources to provide the services clients demand, this leads them to depend on external resources, such as strategic alliances, which can put service infusion into practice with the exchange of resources or capacities between the parties (Kowalkowski et al. 2013). The servitization strategy implies that firms implement cultural changes in their whole organisational structure (Gebauer et al. 2012), but the lack of competences, organisation and resources prevents developing the strategy (Man et al. 2002). The contributions generated by strategic alliances allow firms to obtain benefits in the form of assets (Todeva and Knobe 2005). Resource management and attribution, however, is carefully considered, but it allows the execution of services and their tangible or intangible nature provides the parties with value (Chalal et al. 2015). Once more, the answers obtained agree with the literature, as strategic alliances allowed Firm C to improve its service through having more material services at its disposal, and Firm D to save by not investing in new resources as the partner already had some. Therefore, strategic alliances mean firms can have more resources at their disposal to create and/or improve their services.

Summarising, strategic alliances can play a very significant role in the servitization of small and medium-sized companies. They stimulate the creation of innovation and allow value creation, with firms being able to develop new, differentiated and diversified services. They provide opportunities to gain competitive advantages that help them to stand out from the competition and collaboration between partners allows the exchange of knowledge, experiences and resources, which are essential to help develop new business models.

## Summary of the empirical evidence

From the empirical evidence obtained, there follows a summarised framework based on four dimensions (Table 5) and the results obtained with strategic alliances for successful servitization. The outstanding motive is value creation, i.e., partners’ contribution to an alliance gives access to more resources and knowledge, can improve the quality of service, standing out from competitors and increasing customer satisfaction. As value creation corresponds to an innovation process, firms can also improve their innovation processes, introducing new provisions and filling gaps in their business models. The participant firms say they have a different and diversified portfolio, once more standing apart from their rivals, thanks to their allies’ contribution which allowed firms to combine resources, avoiding investment in material to carry out a service. The main revelations from the interviews are presented in Table 5.

**Table 5:** Main findings in the literature and the interviews. *Source*: Own elaboration from the literature and interviews

Dimension	Results in the literature	Results from the interviews
Main reasons	Value creation	Value creation
	Business development	Firm development
	Obtain more resources	Obtain more resources
Main results	Value creation	Value creation
	Sharing knowledge and costs	Innovation
	Exploiting new resources	Differentiation/diversification

There was seen to be agreement between the literature and the interviews, as SMEs’ reasons are mainly to obtain more resources and develop their business in order to create more value. Although they face some difficulties, such as defining common objectives and running the risk of their firms being taken over, the results are fundamentally positive, since they confirmed increased quality and new forms of innovation and differentiation, creating more value for the companies.

## Conclusions

This study sought principally to understand in greater depth how infusing products with service via strategic alliances among SMEs, aiming for successful servitization. To achieve this, a qualitative approach was adopted, through multiple case studies of three Portuguese SMEs and three Spanish SMEs. Data were collected through semi-structured interviews, following a script that was applied to the SME owners–managers.

The results obtained show that the main reasons indicated by the SMEs for resorting to alliances were the possibility of creating value, obtaining more resources and developing their business more. Based on this study, it is also concluded that servitization is an innovation strategy, and strategic alliances can be said to play a significant role in that type of process, principally in the SME context. Indeed, this firm segment finds it more difficult to develop servitization processes and forming alliances can help them to obtain resources and results that they might not reach alone or would take longer to achieve.

### Theoretical and practical implications

This study presents theoretical and practical implications. From the theoretical point of view, this study contributes to theory in these two areas of knowledge (servitization and alliances), by showing in depth how forming alliances between SMEs can facilitate the introduction of services to their business models. It also shows the competitive advantages that SMEs can extract from those alliances. i.e., the results they can obtain for their business (value creation) in the field of servitization.

**Fig. 1:**
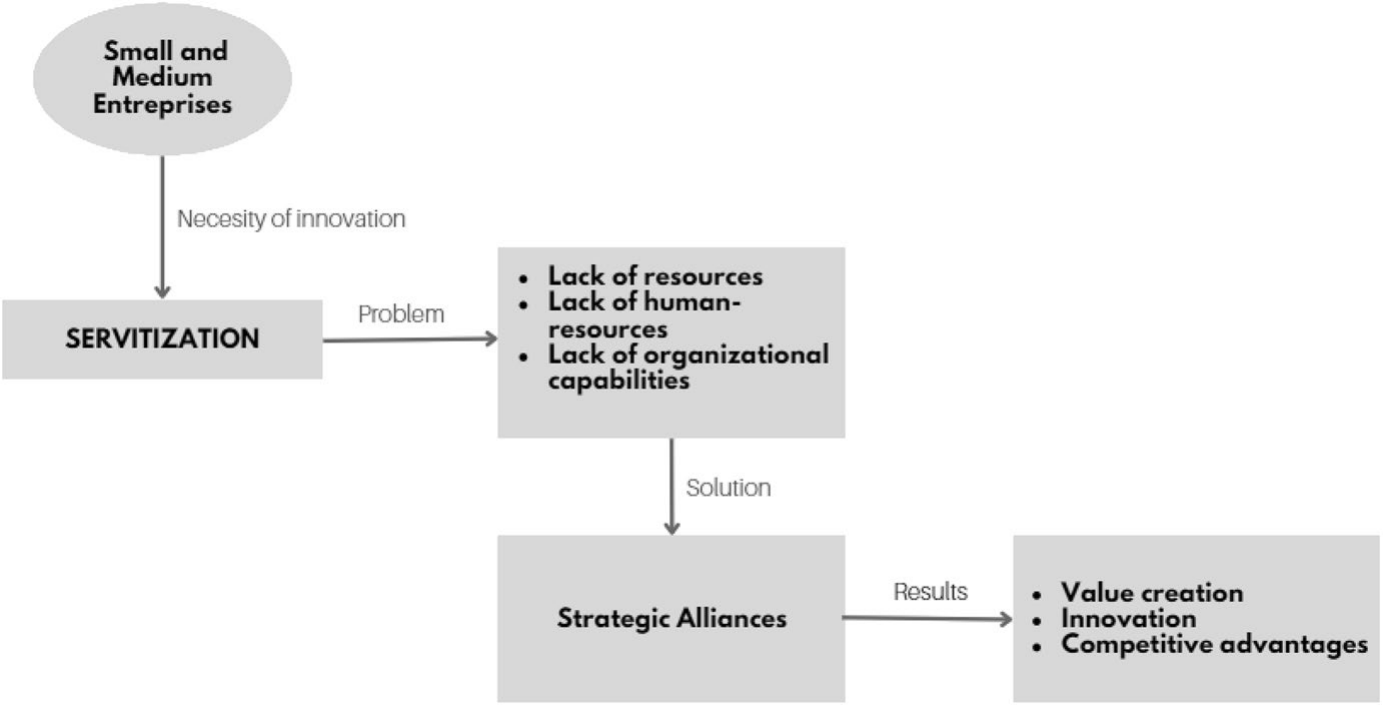
Model proposal for SMEs servitization

The formation of strategic alliances can be seen as “driver” and “enabler” of the servitization, because it can help the SMEs’ overcome barriers, such limited financial resources by sharing costs, or develop projects together from mutual contributions. Therefore, from our empirical evidences, we present a model (Fig. 1) for SMEs’ managers, since it can help define strategic planning and in understanding the impact of these two strategies: strategic alliances and servitization of cooperation. Moreover, the results obtained can form a benchmarking process for SMEs in general, concerning strategic alliances as a mechanism for their successful SME servitization, aiming to improve the functions and processes of a given enterprise.

From a practical perspective, this study also suggests that manufacturing or service SMEs should try to innovate in their business models. SME owners–managers should invest in the servitization strategy, i.e., introduce new provisions in their business models, by introducing services. In this way, these small companies find it easier to add value, both for their activities and for their final customers. Servitization will reinforce the firm’s competitive position in its respective market, by introducing something innovative and different, standing out from its rivals. However, embarking on this servitization strategy involves investment in resources and a change in culture, which can have implications for the whole business and the way of operating. The development of management skills and the possibility of gaining experience through mutual contribution or simple observation is valuable, as it allows parties to obtain private benefits when they start operating in their business models, as well as better organizational performance.

SMEs have some organisational limitations regarding the construction of a different culture—one oriented to service. It is therefore recommended that SMEs form alliances with other firms (from the same sector or with different activities), as a way to achieve successful servitization. Forming strategic alliances originates and strengthens new competitive advantages, such as access to additional resources, new technology and knowledge, which can help SMEs to diversify their business and overcome barriers by distributing risk and keeping costs under control. Therefore, the model proposed here shows that strategic alliances and servitization are linked strategies and are complementary to each other.

These considerations also represent insights for SMEs involved in managing servitization processes. The results of this study aim to go deeper into the contributions of strategic alliances between servitized SMEs, so that they can be successful and sustainable. The conclusion can also help firms that decide to implement the servitization strategy. They may turn to strategic alliances, knowing the motives, benefits, challenges and risks, and essentially, the main results obtained.

### Limitations and future research needs

As with any study, some limitations are implicit and future studies are need. For example, productivity with poor performance, opportunism, knowledge leakage and disparate contributions (Gulati et al. 2012) or even lack of commitment and cooperation (Franco and Franco 2017) can be some of the challenges/risks that an alliance can face. According to Spieth et al. (2021), the main challenge of alliances is the ability of stakeholders coordinate activities and cultures into one, even more when pursuing a collaborative innovation strategy. By discover the main general challenges and risks of these cooperation relations, can help the managers prevent these situations. Therefore, more studies should be realized to identify these challenges and/or risks in strategic alliances for servitized firms.

Service transition must be something initiated from the organizational culture, where each company should review their own culture in order to identify issues when decided to change it (Kinnunen 2011). Therefore, each organizational culture, i.e., service transition variates from company to company, as the identity and the core focus of each one of them are different (Josephson et al. 2016). The transitions to services models are followed by cultural reorganizations and managers understand that is not always linear (Lexutt 2020), however, do they understand that it is required to organize a structure and develop new capabilities, especially when it is passed from a product base-model to a service base-model? In addition, the level of understanding in SMEs regarding service transition is need for further studies.

The creation and delivery of value to customers has become, in the past years, increasingly complex and uncertain, where the ones who can understand the needs, usage and interactions of the product/service with the customer, can benefit from it (Khanra et al. 2021). The customer satisfaction and the way they interact with the service must be measure, i.e., the companies should develop metrics to understand the needs of their customers in order to expand the business and create credibility and reputation in the market (Rodrigues et al. 2021). Looking to the customer as a partner can help firms to develop customized solutions, therefore build a customer-centricity service can support the service strategy and bring results (Kinnunen 2011). This study considered the firm perspective, in order to identify the main variables and dimensions selected and how these affect business models in creating value. However, it did not include analysis of information from the customer’s perspective, which could identify new challenges and risks, as well as presenting other results. Future studies can show empirically how customers insights can change the course to follow. It is suggested therefore that future studies on this topic should also include clients’ opinions.

Define a new business model requires an internal re-organization (Chalal et al. 2015). According to Franco (2011), firms select intentionally their strategic partner, by their business activity, knowledge of market, resources and/or personality of the entrepreneur. The literature about criteria to choose partners appears to be well filled on this topic and agrees to be a fundamental element of alliances success (e.g., Hoffer 2020). However, few studies have explored the way these alliances are managed. The existing literature recognize that alliances can fail when there is no trust and communication, or low commitment (Hofer 2020), therefore, Das and Teng (2000) suggest the creation of governance mechanisms to protect these from potentials risks. The same authors give the example that owners enjoy clear property rights over certain resources (human, financial, physical) or rights to use the resources (patents, contracts, brands), so that others cannot take them away without their consent, and establishing a contract will help the companies be protected from potential interests.

The role of digital transformation in SME management is seen as extremely important for these firms’ future success and to attain competitive advantages. SME managers ascertain the influence digitalization can have on their firms’ management, allowing them to take advantage of and implement some easily the strategic alliances developed. It shows some of the advantages of digital transformation that SME managers can, and should, take into account. It also demonstrates to SME managers, in various sectors of activity, how the adoption of new technology (e.g., social networks) can bring major advantages and contribute to their firms’ success. Studies such as FFMS (2019) or Gebauer et al*.* (2020) went deeper into servitization in the digital era, which is a recent development in this strategy, allowing new forms of value creation. Another future line of research in the area of servitization could consider the adoption of digital technology, for example, Facebook or Amazon, with the possibility of creating markets online. Future researchers could go deeper into the concept of smartization or digital servitization, which combines the concept of servitization in a digital context, i.e., through social networks.

Despite enabling the development of competitive advantages, the transition to a service business model brings changes within and outside organisational boundaries, such as, organisational conflicts and loss of focus (Benedettini et al. 2015). These barriers that SMEs face are more common to larger firms (Rapaccini et al. 2019). Matthyssens and Vandenbempt (2008) identify the lack of organizational capabilities and the acceptance of the strategy by company members as internal barriers, and the individual customizations of products to each customer as external barriers. In turn, Xu et al. (2021) identify as internal barriers, the lack of resources, technologies, manpower and knowledge, and as external barriers, the costs of raw materials and labor costs. However, these barriers can be more important in SME context.

On the other hand, the Innovation Ecosystem refers to all type of innovation, such economic, social, organizational, among others, which is not confined to technological innovation and come as a reaction to the value capture and competitive focus (Granstrand and Holgersson 2020). In addition, one positive element linked to the Innovation Ecosystem is the learning orientation for new capabilities (Imanto et al. 2019). The same authors stated that innovation ecosystem model contributes for the development of innovation capabilities of SMEs, such organization structure, leadership culture or ability to transform knowledge and ideas into products or services. As seen in this study, strategic alliances can improve innovation processes of SME, however, more studies should explore the benefits of these two strategies to reinforce the arguments presented.

For Lütjen et al. (2017), future studies should focus more on the biases and restrictions that may be inherent to non-manufacturing firms in the matter of service transition. Although previous studies consider service transition as a resource for SMEs struggling with smaller and smaller market margins, due to competitive equality in many segments, identifying the barriers associated with servitization in non-manufacturing firms can be a valuable contribution to managerial practice. Thus, further studies can even be realized to show the consequences of implementing advanced services in non-manufacturing firms, as well as on the growth opportunities afforded through servitization in public service firms.

Consequently, during this work, a model was proposed for SMEs. One future research could explore the opportunity to test the findings of this study on a company that wants to innovate it business model, i.e., introduce the company to the servitization strategy and guide them to search a strategic alliance, in order to observe and analyse the results. Thus, future research about servitization is necessary to explain the challenges in this research area and which trend topics require more effort from academics. Against this backdrop, Table 6 displays some research questions (RQ) for a future research agenda.

**Table 6:** Future research questions

RQ1: Which the challenges and/or risks in strategic alliances for servitized firms?	RQ2: What is the level of understanding in SMEs regarding service transition?
RQ3: Customers have a key role in servitization, therefore, how can customers’ insights bring benefits to the enterprise?	RQ4: How the strategic alliances are organized and managed in servitization contex?
RQ5: How potential partners in strategic alliances are selected for servitization?	RQ6: How digital transformation and/or social networks can explain SMEs’ responses to servitization?
RQ7: What barriers are faced by SMEs, preventing them from developing service transition?	RQ8: Being part of an Innovation Ecosystem, how can SMEs benefit from this strategy?
RQ9: Which the consequences of implementing advanced services in non-manufacturing firms?	RQ10: How growth opportunities afforded through servitization in public service firms, can expand and improve their service provision and profit margins in commodity markets?

In relation to the research methods, our empirical study was centred on only two regions/two countries, and a sample of only six SMEs. These firms operate in different areas, and if firms from the same area had been selected, the results could have been different. Indeed, convenience samples should not be generalized, since the exploration is less (Freitag 2018). At first sight, this situation may be considered a disadvantage of this study, but it is important to highlight that the requirements for this research were met and similar results were obtained to those found in the literature review, reinforcing previous research and contributing new insights. Even so, future studies could consider larger firm samples and adopt quantitative methodologies, involving other regions of the world.

Despite these limitations, it is hoped that this study will encourage other researchers to continue investigating this area of knowledge and encourage SME owners–managers to adopt servitization as an innovation strategy, using strategic alliances as a tool to accelerate the process.

## Data Availability

There is no data availability.
